# Mitigation of the Negative Effect of Drought and Herbicide Treatment on Growth, Yield, and Stress Markers in Bread Wheat as a Result of the Use of the Plant Growth Regulator Azolen^®^

**DOI:** 10.3390/plants13162297

**Published:** 2024-08-18

**Authors:** Sergey Chetverikov, Elena Kuzina, Arina Feoktistova, Maxim Timergalin, Timur Rameev, Margarita Bakaeva, Gleb Zaitsev, Alexandr Davydychev, Tatyana Korshunova

**Affiliations:** Ufa Institute of Biology, Ufa Federal Research Centre, Russian Academy of Sciences, Ufa 450054, Russia; che-kov@mail.ru (S.C.); misshalen@mail.ru (E.K.); feoktistova.arisha@yandex.ru (A.F.); timermax@mail.ru (M.T.); rameevt@mail.ru (T.R.); margo22@yandex.ru (M.B.); shur25@yandex.ru (A.D.); korshunovaty@mail.ru (T.K.)

**Keywords:** wheat, biopreparation, herbicide, 2,4-D, drought, antistress agents

## Abstract

Most chemical pesticides, in addition to their main functions (protection against diseases, weeds, and pests), also have a noticeable inhibitory effect on target crops. In a laboratory experiment and two-year field experiments (Russia, Trans-Urals), a study was made of the effect of the biopreparation Azolen^®^ (*Azotobacter vinelandii* IB-4) on plants of the Ekada 113 wheat variety under conditions of drought and stress caused by the exposure to the herbicide Chistalan (2.4-D and dicamba). The biopreparation and the herbicide were used separately and together on wheat during the tillering phase. Treatment with the biological preparation under stressful conditions had a significant effect on the hormonal balance of plants (a decrease in the amount of abscisic acid and a normalization of the balance of indolyl-3-acetic acid and cytokinins in shoots and roots of plants was noted), while the osmoprotective, antioxidant, and photosynthetic systems of plants were activated. In drought conditions, the treatment of plants with biological preparation prevented the inhibition of root growth caused by the use of the herbicide. This, in turn, improved the absorption of water by plants and ensured an increase in wheat yield (1.6 times). The results obtained give reason to believe that microbiological preparations can be used as antidotes that weaken the phytotoxic effect of herbicidal treatments, including in drought conditions.

## 1. Introduction

Despite the global trend toward the greening of agriculture, modern crop production still needs chemical crop protection agents [1]. Among them, the most numerous class (48% of the global consumption of pesticides [2]) are herbicides that are used to destroy weeds. However, in addition to their main function, these agents can cause stress, even in tolerant varieties. This is manifested in a decrease in seed germination, a slowing down of growth and various metabolic processes in plants, and a decrease in their resistance to diseases, which, ultimately, leads to a drop in yield. Sometimes, this negative effect is expressed in the form of obvious morphological signs (necrosis, leaf curl, burns, etc.) [3] It is believed that the death of plant tissues treated with herbicides, such as 2,4-D, is caused by oxidative stress caused by increased production of various reactive oxygen species (ROS) [4,5]. ROS cause damage to most biological molecules and death of plant cells. Antidotes (antistress agents) help to neutralize herbicide stress. They reduce the toxicity of herbicides to cultivated plants and, at the same time, do not affect their activity against weeds [6]. Until recently, chemical compounds were used as antidotes, which bind and block the entry of herbicides into cultivated plants, promote their decomposition in tissues, stimulate metabolic processes, etc. The synthesis of such substances is quite complicated and significantly increases the cost of pesticides. In addition, most antidotes only work with certain herbicides and plant species and, under certain conditions, can have their own toxic effect on the environment [7]. Biological antistressants, such as microorganisms, are deprived of this disadvantage. Herbicide-resistant bacteria with PGP properties, which are capable of stimulating plant growth, increasing their yield in the presence of herbicides, and accelerating the decomposition of these substances in the soil, have been previously described [8,9]. A number of studies have attempted to explain how PGPB affects plant resistance to herbicides. It is assumed that the mechanism of the protective action of *Bacillus megaterium* 501^rif^ bacteria in relation to the herbicide prometrin is due to the positive effect of their metabolic products, in particular, poly-beta-hydroxybutyric acid, on corn (*Zea mays* L.) and oat (*Avena sativa* L.). In addition, these microorganisms are actively involved in herbicide degradation [10]. It has been shown that the bacteria *Serratia rubidaea*, *Pseudomonas putida*, *Serratia* sp., *Synorhizobium meliloti*, and their combinations stimulate the growth of alfalfa (*Medicago sativa* L.) and increase their resistance to imazethapyr-induced herbicide stress using antioxidant enzymes [11]. It has been suggested that endophytes can directly contribute to herbicide detoxification due to their ability to metabolize xenobiotics in plant tissues [12].

Currently, in the context of global warming and climatic instability, crops in many countries are increasingly suffering from water scarcity [13,14]. Drought is considered a key factor that reduces yield [15]. Growth-promoting bacteria can be used to level the stress caused by lack of moisture [16,17]. They are considered an inexpensive and environmentally friendly tool for increasing the adaptive potential and productivity of plants in changing environmental conditions, as well as for restoring soil health [18,19]. Some PGP-bacteria can directly influence plant adaptation to drought through the production of phytohormones, osmolytes, antioxidants, volatile compounds, exopolysaccharides, and 1-aminocyclopropane-1-carboxylate deaminase [20,21]. These metabolites promote water uptake and retention by plant tissues, counteract oxidative stress, and limit the production of stress hormones. Thus, there is quite a lot of research on the effect of PGPB on the adaptation of plants to water deficiency or to the presence of herbicides. However, in real field conditions, plants experience these types of stress at the same time. The combination of herbicidal treatment and a lack of moisture leads to an increase in damage compared to the effect of these negative factors separately [22,23]. There are few publications describing the positive effect of microorganisms on the resistance of crops to drought and herbicide stress [22,23], and we did not find any works that studied the effect of growth-promoting bacteria on the level of hormones in cereals with such combined stress. It would be of particular interest to investigate this effect in relation to phytohormonal herbicides (2,4-D, dicamba).

Earlier, the microbiological fertilizer Azolen^®^, based on the strain of nitrogen-fixing bacteria *Azotobacter vinelandii* IB-4, was developed at the Ufa Institute of Biology of the Ural Federal Research Center of the Russian Academy of Sciences. The biopreparation increases the resistance of vegetable and cereal crops to diseases and, as a result, provides a high yield of plants within their volume ability [24,25,26,27]. At present, Azolen^®^ is produced by several enterprises on an industrial scale and is actively used in various climatic zones of Russia. Previously, a positive effect of this biopreparation in a tank mixture with an auxin herbicide on the growth parameters of wheat under moderate watering in a laboratory experiment and on its yield under field conditions in a non-drought year was shown [28]. However, there are no scientific data on its effect on the productivity and yield of agricultural plants under the combined influence of herbicides and drought. We assumed that the presence of the biopreparation Azolen^®^ phytohormonal activity would help wheat (*Triticum aestivum* L.) overcome the effects of complex stress (normalizing hormonal status, compensating for growth lag, and stabilizing the water balance). Ultimately, this would lead to an increase in the yield of this crop.

The purpose of this work is to study the effect of the biopreparation Azolen^®^ on morphometric and biochemical parameters and the hormonal status of wheat plants, as well as to evaluate the effectiveness of its use to increase its yield with insufficient moisture and spraying with the auxin-like herbicide Chistalan.

## 2. Results

### 2.1. The Influence of Treatments on the Morphometric Characteristics of Plants, Biochemical Parameters, and the Level of Evapotranspiration in the Laboratory Experiment

#### 2.1.1. Evapotranspiration

**Figure 1:**
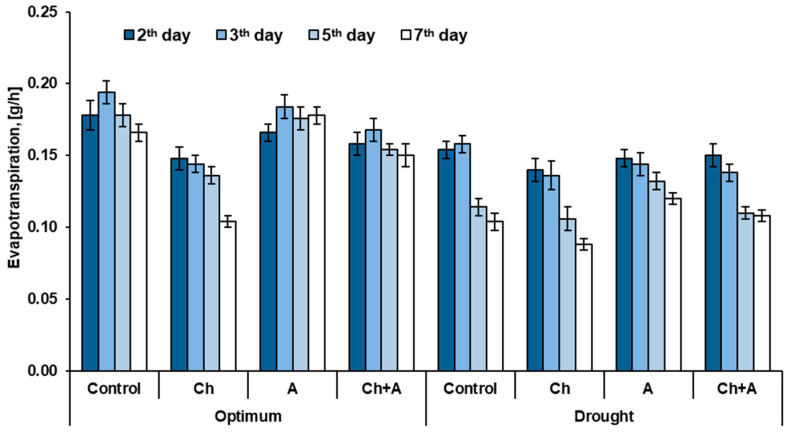
Dynamics of evapotranspiration per plant after wheat treatment, *n* = 30. Optimum: 60–80% of soil moisture capacity; drought: 30–40% of soil moisture capacity; control—no treatment was performed; Ch—Chistalan treatment; A—Azolen^®^ treatment. Data are presented as mean ± SE, *p* ≤ 0.05, *n* = 30.

Herbicide treatment significantly reduced the evapotranspiration of wheat plants (Figure 1). On the 3rd and 5th days after spraying with Chistalan, this indicator differed from the control by 1.3, and on the 7th, by 1.6 times. At the same time, plants treated with a mixture of the herbicide and the biopreparation showed a tendency to increase evaporation compared to the variant where only the herbicide was used.

With insufficient soil moisture, the level of evapotranspiration was significantly reduced in all variants of the experiment. At the same time, it was found that under drought conditions, plants inoculated with Azolen^®^ performed better than untreated plants or plants sprayed with the herbicide. This is evidenced by the fact that under conditions of artificial drought, the level of plant transpiration on the 7th day after the application of Azolen^®^ was higher than that of control plants by 15%.

#### 2.1.2. Hormone Content

**Figure 2:**
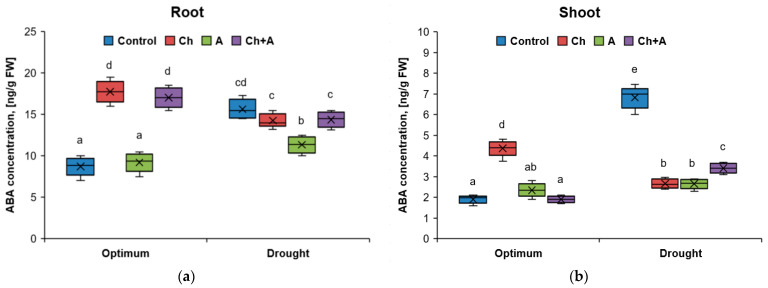
The amount of abscisic acid in wheat roots (**a**) and shoots (**b**) on the 3rd day after treatment. Optimum—60–80% of soil moisture capacity; drought—30–40% of soil moisture capacity; Control—no treatment was performed; Ch—Chistalan treatment; A—Azolen^®^ treatment. Data are presented as median and quartiles. Statistically different means are indicated by different letters (*p* ≤ 0.05, *n* = 6).

In a laboratory experiment, the effect of various types of abiotic stress (herbicide, drought, and herbicide + drought) on the content of hormones in the shoots and roots of wheat plants was studied. In variants with sufficient moisture, three days after spraying the plants with the herbicide solution, the content of abscisic acid (ABA) in the roots increased by 1.9–2.0 times (Figure 2). The response of wheat to drought was comparable to the herbicide treatment. The combined action of two stress factors did not provoke an increased accumulation of ABA in the root system, compared with the action of each of the stress factors separately. The content of abscisic acid in the shoots under stress increased more significantly than in the roots: by 2.2 times after the herbicide treatment and by 3.6 times under the influence of drought.

Spraying plants only with the biopreparations Azolen^®^ with sufficient watering did not lead to an increase in ABA synthesis, that is, the introduction of microorganisms was not perceived by plants as a stressful effect. The simultaneous treatment of plants with the herbicide and the bacteria led to a decrease in the accumulation of ABA in the shoots compared to plants treated with the herbicide alone.

Against the background of drought, spraying with the biopreparations of Azolen^®^ contributed to a decrease in the ABA content in the roots by 1.4 times, but the plants did not react in this way to the presence of Azolen^®^ in a mixture with the herbicide.

**Figure 3:**
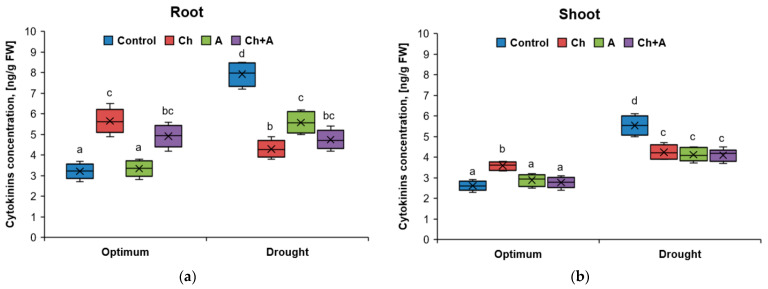
The amount of cytokinins in wheat roots (**a**) and shoots (**b**) on the 3rd day after treatment. Optimum—60–80% of soil moisture capacity; drought—30–40% of soil moisture capacity; Control—no treatment was performed; Ch—Chistalan treatment; A—Azolen^®^ treatment. Data are presented as median and quartiles. Statistically different means are indicated by different letters (*p* ≤ 0.05, *n* = 6).

A similar pattern was established for cytokinins. After the use of the herbicide, their content in the roots increased by 1.7 times, and in the variant with a moisture deficit, by 2.5 times. Similarly, under the influence of unfavorable factors, this hormone increased in plantshoots (Figure 3). The introduction of Azolen^®^ under normal moisture did not affect the content of cytokinins in any way; against the background of drought, their concentration decreased both in the shoots and in the roots by 1.4 times. The addition of Azolen^®^ to the herbicide under optimal moisture conditions resulted in a 1.3 times decrease in cytokinins in the shoots. With water deficiency, the microorganisms in the presence of the herbicide did not have a significant effect on the content of this phytohormone.

**Figure 4:**
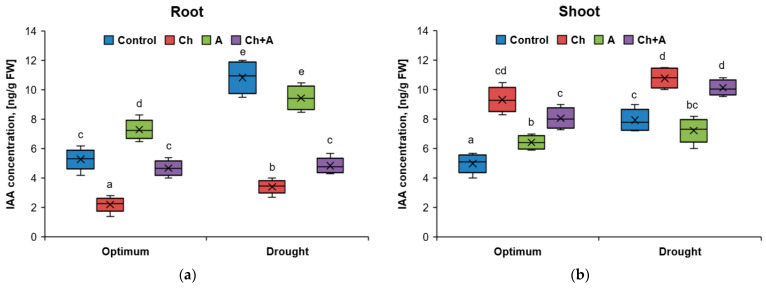
The amount of indolylacetic acid in wheat roots (**a**) and shoots (**b**) on the 3rd day after treatment. Optimum—60–80% of soil moisture capacity; drought—30–40% of soil moisture capacity; Control—no treatment was performed; Ch—Chistalan treatment; A—Azolen^®^ treatment. Data are presented as median and quartiles. Statistically different means are indicated by different letters (*p* ≤ 0.05, *n* = 6).

Drought caused the accumulation of indolyl-3-acetic acid (IAA) in the roots, but only in those variants where the herbicide was not used. Treatment with Chistalan led to an increase in the concentration of IAA in plant shoots. Compared to the control, its content increased by 1.8 times under conditions of normal moisture and by 1.4 times under moisture deficiency (Figure 4). This resulted in an imbalance in the distribution of auxins between the shoots and roots of plants. In the absence of stress factors, treatment with the bacteria contributed to an increase in the concentration of IAA in shoots and roots of wheat. When using the biological preparation together with the herbicide, the distribution of IAA between the aboveground and underground organs tended to be close to the parameters of the plants that were not subjected to stress.

#### 2.1.3. The Content of Malondialdehyde, Proline, and Chlorophyll

**Figure 5:**
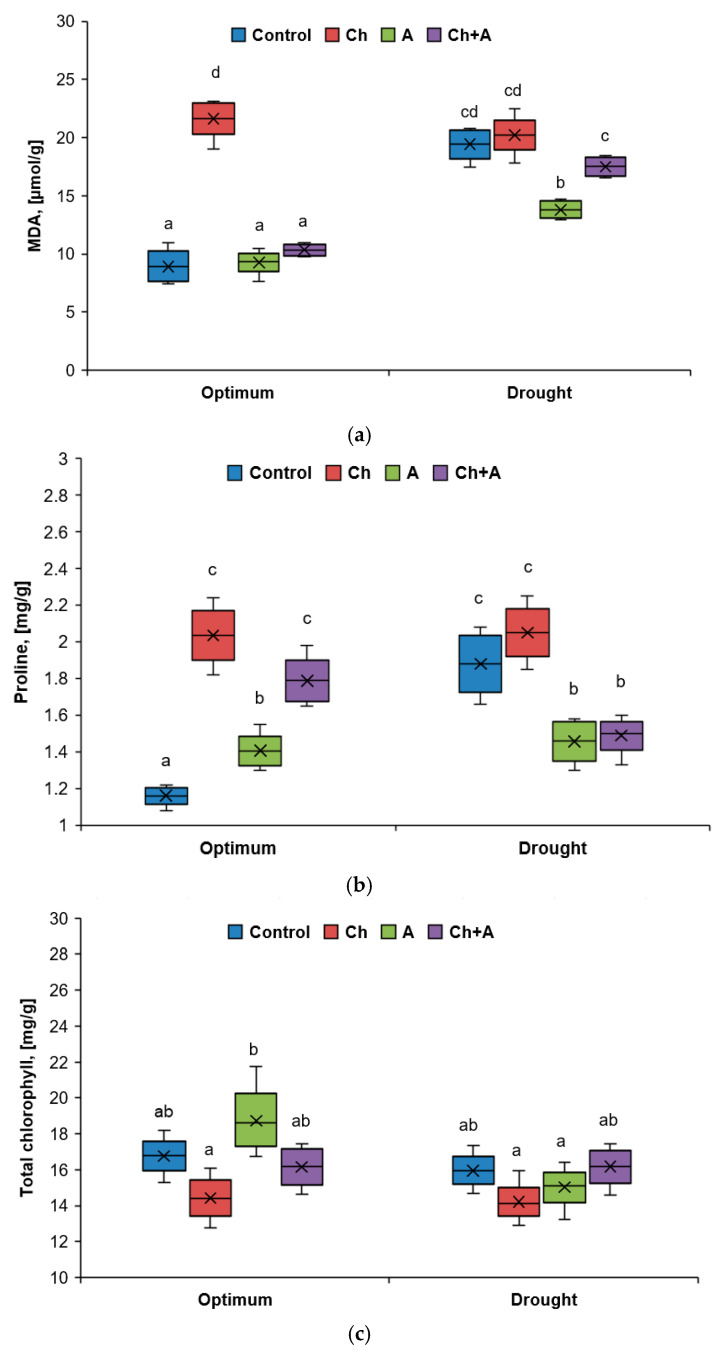
The amount of malondialdehyde (**a**), proline (**b**), and chlorophyll (**c**) in wheat leaves on the 3rd day after treatment. Optimum—60–80% of soil moisture capacity; drought—30–40% of soil moisture capacity; Control—no treatment was performed; Ch—Chistalan treatment; A—Azolen^®^ treatment. Data are presented as median and quartiles. Statistically different means are indicated by different letters (*p* ≤ 0.05, *n* = 9).

In wheat plants, the amount of malondialdehyde (MDA) in the leaves increased when they were sprayed with the herbicide and under conditions of artificial drought (Figure 5a). When exposed to the biopreparation, MDA accumulation did not occur. The addition of the bacteria to the herbicide with sufficient watering led to a decrease in the amount of MDA in the leaves to the control values. The treatment of plants with the biopreparation Azolen^®^ against the background of stress caused only by drought contributed to a decrease in the amount of MDA by 1.4 times. A similar effect was absent if the drug was used under combined stress (the herbicide + drought).

With a deficit of soil moisture and/or spraying with the herbicide, the concentration of proline in the shoots of plants increased sharply (Figure 5b). The treatment of plants with the biopreparation Azolen^®^ also led to an increase in this indicator, although the accumulation of proline was less significant. During drought, under the influence of the bacteria, a decrease in proline occurred, including in the presence of the herbicide.

The content of chlorophyll is also an important indicator of the state of plants under stress. When treated with the herbicide, the total content of chlorophylls a and b decreased (Figure 5c). The biopreparation, alone and in combination with the herbicide, apparently contributed to the improvement in the plant condition, but no significant increase in the amount of photosynthetic pigments was found in its presence.

#### 2.1.4. Morphometric Indicators

**Figure 6:**
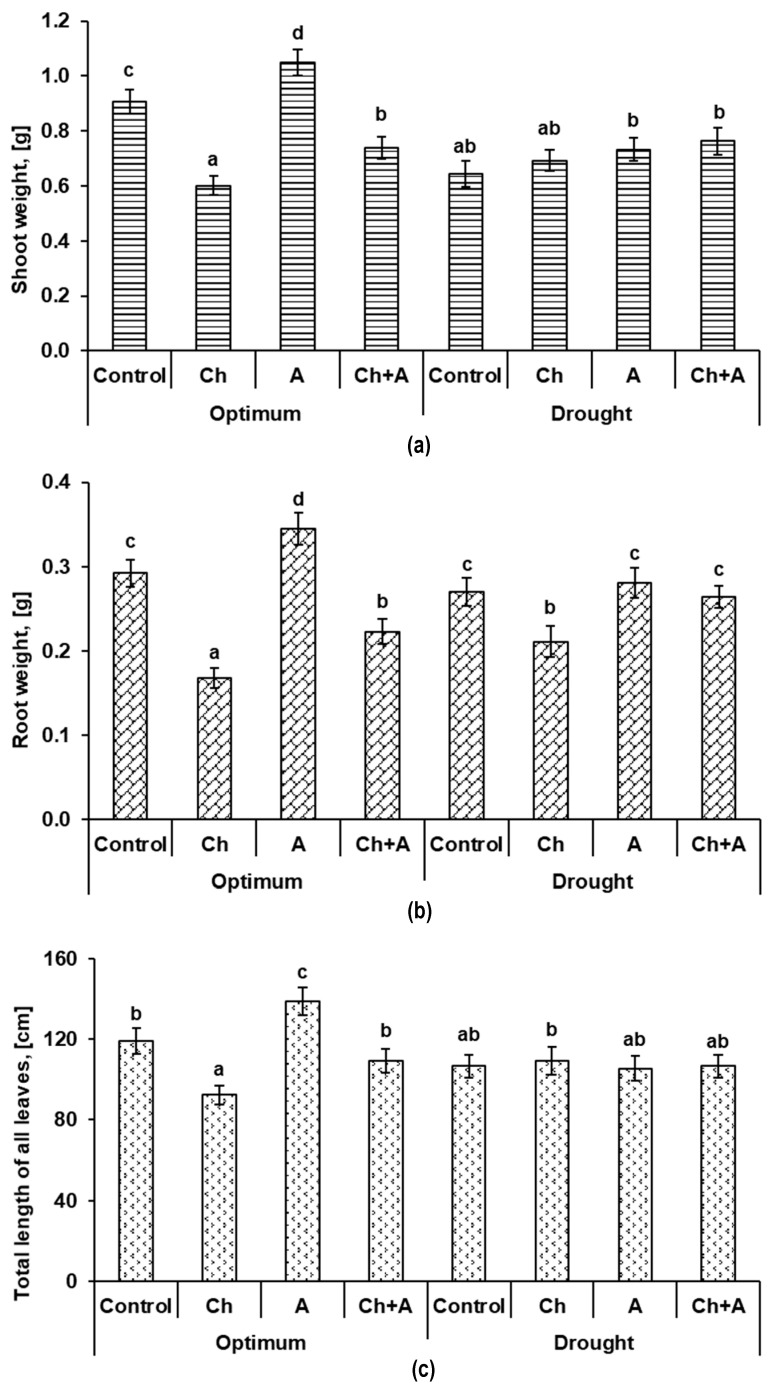
The weight of shoots (**a**), roots (**b**), and total length of all leaves (**c**) of wheat plants on the 14th day after treatment. Optimum—60–80% of soil moisture capacity; drought—30–40% of soil moisture capacity; Control—no treatment was performed; Ch—Chistalan treatment; A—Azolen^®^ treatment. Data are presented as mean ± SE. Statistically different means are indicated by different letters (*p* ≤ 0.05, *n* = 50).

The morphometric characteristics of plants obtained fourteen days after treatment indicate that under the influence of the stress factors, the mass of shoots in wheat plants decreased by 1.3–1.5 times (Figure 6a). A synergistic effect was not found under the complex effect of the stress factors. On the contrary, the smallest mass of plant shoots was recorded in the variant where the herbicide was used, but the plants grew under conditions of normal moisture supply.

The use of the biopreparation Azolen^®^ together with the herbicide (in the absence of drought) did not completely prevent the inhibition of the growth of plant shoots, but significantly reduced its rate. With a lack of watering, the mass of shoots for all variants of the experiment was 0.644–0.763 g versus 0.907 g in the control with a normal level of moisture. The use of Azolen^®^, including in a mixture with the herbicide, did not have a significant effect on plant growth.

By the time the leaf length measurements were taken, five true leaves had fully unfolded. The smallest total length of leaves, by analogy with the mass of shoots, was found in plants treated with the herbicide (Figure 6c). The impact of two adverse factors (the herbicide + drought) turned out to be less harmful for plants than herbicide treatment under normal watering conditions.

Despite the fact that during drought (without additional treatments) the mass of shoots significantly decreased, the mass of roots almost did not change (Figure 6b). On the other hand, when treated with the herbicide (under conditions of normal soil moisture), there was a significant decrease in the weight of the roots (by 1.7 times). The use of the biopreparation simultaneously with the herbicide had a positive effect on the growth of roots in both variants of moistening. The mass of roots increased by 25.1–32.7% due to the addition of Azolen^®^ to the herbicide as compared to the pure herbicide treatment.

Thus, in a laboratory experiment, it was found that the herbicide stress turned out to be more devastating for plants than the lack of sufficient water. Under the combined stress (the herbicide + drought), no noticeable deterioration in plant condition was observed compared with the influence of the herbicide and drought separately. In the variants where Azolen^®^ was used together with the herbicide, the mass of shoots and roots, as well as the length of the leaves of the plants, was greater than when treated with the herbicide alone. Thus, the biopreparation helped plants cope with the stress caused by the presence of chemicals toxic to them.

### 2.2. Influence of Treatments on Growth, Biochemical Parameters, and Yield of Wheat in the Field

#### 2.2.1. Characteristics of Water Relations

The results of the laboratory experiments indicated that the biopreparation Azolen^®^ enhances plant evapotranspiration during drought, while the herbicide suppresses it (Figure 1).

To test the effect of various treatments on plant water consumption under dry field conditions, the relative water content (RWC) was measured in wheat leaves after they were sprayed with the biopreparation, the herbicide, or a mixture thereof. After the application of Chistalan, RWC was 10% lower than in untreated areas (Table 1). Under conditions of a combination of herbicide stress and drought, Azolen^®^ contributed to the normalization of water consumption to the control values.

#### 2.2.2. The Content of Hormones in Shoots

The use of the herbicide Chistalan reduced the content of ABA by more than 1.5 times, and the use of the biopreparation Azolen^®^ (alone and in combination with the herbicide) reduced the content of ABA by 1.2 times. All treatment variants led to a sharp increase in the content of IAA in the shoots (by 2–3 times), especially when using the auxin-like herbicide (Table 1). The use of Azolen^®^, including together with Chistalan, led to an increase in the level of cytokines in plants by 1.4 times. In general, the data obtained in the field experiments on the content of ABA and IAA in wheat shoots two weeks after spraying with preparations confirm the trends in their distribution found in the laboratory experiments on the third day after treatment. As for cytokinins, treatment with the herbicide and the biopreparation undoubtedly influenced their production by plants. However, the data obtained do not allow us to unambiguously characterize the nature of this influence.

#### 2.2.3. Chlorophyll and Malondialdehyde Content

In the field experiment, all types of treatment had a positive effect on the content of chlorophyll in wheat plants. This indicator increased especially noticeably (by 1.5 times) when the herbicide Chistalan was used. The biostimulant Azolen^®^ against the background of drought contributed to a 1.5 times decrease in the MDA content in leaves, including when plants were sprayed with the herbicide (Table 1). At the same time, under laboratory conditions, bacterization led to a decrease in the level of this marker of oxidative stress only in the absence of Chistalan.

#### 2.2.4. Growth Indicators, Crop Structure, and Yield of Wheat

The use of the herbicide had a negative effect on the growth characteristics of wheat shoots. At the end of the experiment, it was found that their dry weight decreased by 1.4 times (26.0%) and their length by 1.2 times (13.4%). Treatment with only the biopreparation increased the length of the shoots but did not lead to an increase in their weight compared to the control plants. However, when using Azolen^®^ on plants sprayed with the herbicide, the weight and length of shoots increased compared to the control plants (by 29.2 and 18.8%, respectively) and the plants treated with the herbicide alone (by 74.6 and 37.3%, respectively) (Table 2).

The number of weeds on the plots with different treatments was as follows (plants m^−2^): without treatment—37 ± 3; only biopreparation—33 ± 4; only herbicide—1.5 ± 0.3; herbicide and biological product—1.8 ± 0.4. Weeds belonged to the species *Cirsium arvense* L. and *Convolvulus arvensis* L.

High yields and correspondingly higher economic benefits are the driving force behind farmers’ motivation to plant crops. The inclusion of the biopreparation Azolen^®^ in agricultural technology contributed to an increase in yield. In the absence of the herbicide treatment, it increased by 545 kg/ha; in plants on plots sprayed with Chistalan, it increased by 1366 kg/ha. The amount of straw increased in all treatment variants, but the best results were also achieved when using the biopreparation (Table 2). The inclusion of the biopreparation Azolen^®^ in agricultural technology contributed to an increase in yield. In the absence of the herbicide treatment, it increased by 545 kg/ha; in plants on plots sprayed with Chistalan, it increased by 1366 kg/ha. The amount of straw increased in all treatment options, but the best results were also achieved when using the biopreparation. To pay back the costs of using Azolen^®^ (USD 10/ha at a price of USD 5/L and a rate of 2 L/ha) with a wheat price of USD 250/ton, a yield increase of 50 kg/ha is enough (in value terms USD 12.5/ha). Additional profit from the application of Azolen^®^ in the variant without the herbicide was USD 126/ha, with the herbicide 332 USD/ha (calculated by multiplying the additional yields obtained as a result of applying the biopreparation by the price). And the economic efficiency from the introduction of the biopreparation Azolen^®^, defined as the ratio of the additional profit received as a result of the use of Azolen^®^ to the costs of its use, was 13.6 and 34.2 without the herbicide and with the herbicide, respectively (i.e., as many times the recovery of biological treatment costs).

Among the indicators of wheat yield, the weight of 1000 grains increased only when Azolen^®^ was used without the herbicide (by 10.5% compared to the control). The number of productive stems significantly reacted to all types of treatment. At the same time, the herbicide had a negative effect on this indicator, and bacterization led to its increase by 34.0 (only the biopreparation) and 55.2% (complex application with the herbicide). Spraying with the herbicide Chistalan or the biopreparation Azolen^®^ equally positively affected the number of grains per spike, but the best results were achieved when they were used together (Table 2).

#### 2.2.5. Grain Quality Indicators

Spraying with the herbicide resulted in a decrease in the protein content in the grain, calculated from the total nitrogen. The introduction of the biopreparation simultaneously with the herbicide increased its amount to control values (Table 2). The Azolen^®^ contributed to an increase in the amount of raw gluten in the grain, regardless of whether it was used alone or in a mixture with the herbicide.

## 3. Discussion

Wheat is a strategically important grain crop that is of great importance in ensuring food security around the world [29]. Drought and its combination with other stress factors, such as herbicides, causes significant damage to agriculture, the food industry, and the economy as a whole [29,30,31]. The use of environmentally friendly approaches that induce the natural defense mechanisms of plants to various stresses and their combinations can improve the growth and productivity of crops [32]. Recently, the use of biopreparations for the protection and leveling of abiotic stresses in cultivated plants has attracted increasing interest [8]. In this work, for the first time, we used the biopreparation Azolen^®^, which contains PGPB and was developed at our Institute and approved for use in Russia, in laboratory and field conditions to level stress in wheat plants caused by drought, an herbicide treatment, and a combination of these factors. The positive effect of this biopreparation, when combined with the herbicide based on 2,4-D, on the growth of wheat at the stage of the third leaf appearance has already been shown. However, the results were obtained with watering (laboratory experiment) and with sufficient rainfall (field experiment). The tank mixture of the biopreparation and the herbicide had a positive effect on growth characteristics and maintenance of RWC but did not contribute to a significant increase in yield [28].

It is believed that cultivated cereals are insensitive to the action of herbicides based on the synthetic auxin 2,4-D. However, if applied before the tillering stage, significant inhibition of plant growth is possible [33,34]. In our laboratory experiments, the treatment of wheat with Chistalan in the tillering phase led to a decrease in the weight of the shoot and root of the plants, as well as the total length of the leaves compared to the control plants that were not sprayed with the herbicide. In contrast, under the influence of drought (without the herbicide), only the weight of the shoot decreased. The introduction of the biopreparation Azolen^®^ (included together with the herbicide) with normal moisture contributed to an increase in all three analyzed morphometric parameters. In the case of reduced watering, the presence of the biopreparation mixed with the herbicide had a positive effect only on root growth.

Under laboratory conditions, an increase in the content of IAA in the shoots of wheat plants was found in all variants of the experiment (relative to the control with normal humidity), especially where the herbicide was used (an increase of 1.8–2.1 times). Perhaps, in this case, the accumulation of IAA could be a consequence of the absorption of exogenous auxins by plants [35]. We noted that in the variants with the herbicide treatment, an increase in the concentration of auxins in plant shoots was accompanied by a violation of their transport to the roots. According to modern concepts, IAA is synthesized mainly in the merisystems of shoots and in young leaves and then transported in a polar way to the basally located tissues of the shoot and to the root [36]. The abnormal accumulation of endogenous auxins and auxin herbicides in the growing tissues of shoots negatively affects root formation and tropism [37]. With the simultaneous use of the biopreparation Azolen^®^ and the herbicide Chistalan, the intake of IAA into the root system of plants was not disrupted, and this, of course, had a positive effect on root growth.

The most well studied adaptive response of plants to stress is the production of ABA [38,39]. However, the action of herbicides based on 2,4-D can also lead to the accumulation of this hormone [4]. In the laboratory experiment, treatment with Chistalan led to a twofold increase in the content of abscisic acid in both roots and shoots. With the combined use of the herbicide and the biopreparation, the level of ABA remained the same high. An indirect cause of ABA accumulation could be an increase in the level of auxins in plants since it is known that auxins contribute to the activation of the synthesis of abscisic acid [40]. Elevated ABA content inhibits plant growth [41], which explains why Chistalan treatment had a depressing effect on wheat. On the other hand, it was shown that under conditions of moderate watering, the introduction of bacteria stabilized the concentration of IAA and, accordingly, contributed to a decrease in the level of ABA [28].

Predictably, the effect of drought should have provoked the synthesis of ABA by plants. Indeed, the concentration of abscisic acid increased by 1.7 times in the presence of water deficiency. But after the introduction of the biopreparation Azolen^®^, the rate of production of this hormone slowed down significantly.

Thus, it was shown that ABA accumulation occurred not only during drought but also during herbicide stress. As a consequence, in both cases, we observed a decrease in the intensity of transpiration, a known reaction associated with stomatal closure, provoked by the accumulation of abscisic acid [42,43]. On the other hand, a decrease in transpiration may be due not only to the closure of stomata but also to the shredding of plant leaves [44]. As noted earlier, in the presented experiment, a significant decrease in the above-ground mass of wheat occurred under the influence of both types of stress.

It is known that bacteria can also affect the level of ABA in plants and thus control their transpiration [45]. In the laboratory experiment with insufficient watering, the biopreparation had a positive effect on the water exchange of wheat plants. In the field experiment, against the background of natural drought, the bacteria that make up the biopreparation were also able to neutralize the negative effect of the herbicide on plants, preventing a decrease in the relative water content in the leaves.

In our work, we also analyzed the content of cytokinins in the shoots and roots of wheat. In our opinion, the role of these phytohormones in increasing the stress resistance of plants to adverse environmental factors is still insufficiently studied [46]. We found that stress led to an increase in the content of cytokinins in both shoots and roots of plants by 1.3–2.0 and 1.7–2.5 times, respectively. When the biopreparation was introduced under conditions of normal moisture, a decrease in the concentration of cytokinins to the control level was observed. With a lack of moisture, their number also decreased in shoots and roots by 1.4 times compared with the control without treatment.

It is known that cytokinins are involved in many physiological processes in the plant, affect the morphogenesis of the shoot and root, and are ABA antagonists [47,48]. It is possible that the accumulation of these hormones under the influence of the studied stress exposure could be caused by the need to compensate for the growth-inhibitory effect of an increased level of ABA. Stomatal closure caused by ABA provoked a number of negative consequences: a decrease in the efficiency of photosynthesis and oxidative and thermal stresses. The role of cytokinins, in this case, consisted of the possible leveling of the negative consequences of reactions aimed at reducing evaporation. It is also possible that cytokinin biosynthesis activated synthetic exogenous auxin 2,4-D [49]. In any case, the increase in their number in plants treated with the herbicides could be one of the reasons for the decrease in root mass [50]. When treated with the biopreparation, the ratio of auxins/cytokinins was close to that observed in plants without treatments under normal watering conditions.

In general, the ratio of auxins to cytokinins and the crosstalk between them are important factors that determine the development of plant tissues [51,52]. Despite the fact that auxins and cytokinins are antagonists to each other, a certain concentration of auxins is required to activate the action of cytokinins [53]. In addition, the effect of cytokinins on plants, like other hormones, is closely related to plant nutrition factors. Under conditions of mineral nutrition deficiency (for example, due to a decrease in the absorption capacity of roots under the influence of drought), the effect of cytokinins on plants may not manifest itself [54]. In the present study, under drought conditions, the content of cytokinins in the roots increased by 2.5 times, but this did not lead to a slowdown in the growth rate of the root system. It is possible that, in this case, there was a short-term increase in the level of cytokinins or inactivated forms dominated among them.

To quantify the discomfort experienced by the experimental plants, stress markers such as MDA and proline were analyzed. The accumulation of precisely these low-molecular-weight compounds is one of the early adaptive responses of plants to the action of stressors of various natures [55,56]. MDA is known to be one of the metabolites of lipid peroxidation, and its accumulation in plants indicates that they are subjected to severe oxidative stress. In turn, the stress hormone ABA controls both antioxidant systems and the synthesis of osmoprotectors; therefore, an increase in its concentration under unfavorable conditions leads to the accumulation of both MDA and proline in plants [57]. The most significant increase in both of these indicators on the third day after plant treatment was established against the background of the herbicide, drought, and combined stress exposure. Inoculation with the biopreparation, together with the herbicide, led to a decrease in the level of MDA and proline. Thus, it can be concluded that bacterization had a beneficial effect on plants, helping to maintain their growth under stress conditions.

The content of photosynthetic pigments decreases under conditions of abiotic stress [58], including the action of herbicides [59]. In the present study, a slight decrease in the total content of chlorophylls *a* and *b* in the leaves of plants treated with Chistalan was recorded, but these data are not statistically significant. It is known that cytokinin hormones have the ability not only to delay the breakdown of chlorophyll but also to accelerate the formation of its precursor, protochlorophyllide [60]. It is possible that an increase in the amount of cytokinins in plants in response to the inhibition of leaf growth under the influence of the herbicides made it possible to maintain sufficient activity of photosynthesis and contributed to greater resistance of plants under stress.

Plants grown under natural soil and climatic conditions are simultaneously exposed to a wide range of biotic and abiotic stresses, each of which can significantly inhibit their growth and development [61]. Therefore, growth-stimulating bacteria that have proven themselves well in laboratory experiments must be tested for their effectiveness in real field conditions. Since, in this case, it is impossible to simultaneously reproduce the insufficient and normal water regime, we focused on the drought and its combination with the herbicide Chistalan and the biopreparation Azolen^®^.

Stomatal closure under stress leads to a decrease in the carbon dioxide concentration, which promotes the formation of reactive oxygen species [62,63] and lipid peroxidation. The latter manifests itself in the accumulation of MDA [56]. Therefore, the high content of MDA in plants during drought and when it is combined with the herbicide treatment in the field (Table 1) can be explained by lower stomatal conductivity, which should have caused oxidative stress. In the laboratory experiment with insufficient watering, the level of MDA decreased only when using the biopreparation. At the same time, in the field experiment, spraying with the biopreparation led to a decrease in this indicator both in the presence of the herbicide and without it.

It is known that an increase in the amount of abscisic acid caused by drought contributes to aging, which manifests itself in a decrease in the concentration of chlorophyll and inhibition of photosynthesis [64]. At the same time, in all treatment variants, we found an increase in the content of cytokinins, which, as is known, has the opposite effect of ABA on stomatal conductance (maintain stomata open) [65,66,67], stimulates photosynthesis, and prevents aging. Therefore, the decrease in the level of stress-induced accumulation of ABA and the increase in the concentration of cytokinins in the shoots of plants treated with the biopreparation, the herbicide, and their mixture, revealed under field conditions, could lead to an increase in the level of chlorophyll (Table 1) and, accordingly, the activity of photosynthesis.

Against the background of natural drought, spraying with the herbicide suppressed weeds. This softened the competition for water resources, improved the supply of wheat plants with water and mineral nutrients, and also led to a decrease in the level of ABA and an increase in the content of cytokinins in shoots. But the combination of these factors did not cause an increase in the growth of the terrestrial part of plants, and, on the contrary, led to its suppression. The use of the biopreparation also reduced the amount of ABA (although to a lesser extent), improved water metabolism, and increased the rate of photosynthesis. In addition, the biopreparation increased the accumulation of cytokines more noticeably than the herbicide. Probably, in the case of the bacterial treatment (especially in combination with the herbicide that suppresses weeds), a synergistic effect was observed, which led to a significant growth of the above-ground parts of plants (Table 2).

For crop production, it is important to determine whether the effects observed at the beginning of the growing season can lead to an increase in the quantity and quality of the crop. It is known that seeds from additional shoots also make a certain contribution to the wheat grain yield. Treatment with the microbial preparation (especially in combination with the herbicide) led to the activation of tillering and an increase in the number of productive shoots, which contributed to an increase in the yield of grain and straw.

At the same time, the positive effect of the use of the biopreparation was not only the increase in yield but also the accumulation of gluten in the grain. There are conflicting data on the effect of bacteria on the amount of protein in the grain, including gluten, which can increase during bacterization [68,69] or remain unchanged [70] depending on the wheat variety and bacterial strain. The presence of gluten gives the dough elasticity, helps it rise during fermentation, and retains its shape. Therefore, the increase in this indicator under the influence of bacteria that form the basis of the biopreparation can improve the quality of baked flour products. At the same time, after spraying with the herbicide Chistalan, the levels of IAA and cytokinins also increased in plants, but their ratio was strongly shifted toward auxins. This led to a decrease in the number of productive stems but did not significantly affect the yield and the amount of gluten in the grain, negatively affecting the protein content in it.

It is important that in the field experiment, stimulation of the development of additional productive stems did not lead to a decrease in the weight of 1000 grains compared to the control. It is known that cytokinins can influence the tillering of wheat plants and, through it, the number of ears and their grain content [71]. In our study, the accumulation of these hormones in the shoots under all types of treatment had a positive effect on the number of grains in the ears.

## 4. Materials and Methods

### 4.1. Materials

The active substance of the microbiological fertilizer Azolen^®^ is the strain PGPB *A. vinelandii* IB-4 (titer 10^9^ CFU/mL), patented in the Russian Federation [72]. The strain has the ability to fix atmospheric nitrogen and dissolve mineral phosphates, exhibits antagonistic activity against phytopathogenic micromycetes, produces phytohormones of the cytokinin class, and synthesizes extracellular polysaccharides [72,73].

The biopreparation is recommended for use on cereals, legumes, greens, technicals, fodder, vegetables, flowers and ornamentals, and fruit and berry crops. It can be used for pre-sowing seed treatment, as well as root and foliar (spraying) plant nutrition. Treatment with Azolen^®^ leads to an increase in seed germination, plant growth characteristics, acceleration of flowering, fruiting, and increased resistance to phytopathogens. Its use allows a reduction in the dose of mineral fertilizers without loss of yield, thereby reducing the chemical load on the soil. In general, Azolen^®^ contributes to increased yields and provides environmentally friendly products.

The biopreparation has been included in the State Catalog of Pesticides and Agrochemicals Permitted for Use on the Territory of the Russian Federation (State Registration No. 597(598)-19-2525-1) since 2008. The Azolen trademark is registered in the State Register of Trademarks and Service Marks of the Russian Federation.

The herbicide Chistalan is selective against dicotyledons (produced by AHK-AGRO LLC, Ufa, Russia) and contains auxin-like active substances 376 g/L of 2,4-D (2-ethylhexyl ether) and 54 g/L of dicamba (sodium salt) [74].

Seeds of bread spring wheat variety Ekada 113 were used. The variety is drought-resistant, recommended for cultivation in the Ural region of Russia, and has good baking qualities.

### 4.2. Laboratory Experiment

The laboratory experiment was conducted in 2020 in the Laboratory of Agrobiology of the Ufa Institute of Biology, Ufa Federal Research Centre. Seeds of wheat were germinated on moist filter paper. Soil mixture (10% washed and sifted sand, 90% soil from the upper arable layer of chernozem) was placed in plastic vessels (height, 16; diameter, 12 cm), and 10 germinated seeds were planted. Fertilizers were not used during the experiment. Vessels were placed on the light box in accordance with a randomized scheme (Table 3).

Plants were grown at a temperature of 22–26 °C under controlled lighting (photon flux density 190 µmol/m^2^/s, a 14 h photoperiod). In the variants without moisture deficit, the soil moisture was maintained at the level of 60–80% of the soil moisture capacity throughout the entire experiment. To simulate drought, soil moisture content was constantly maintained at the level of 30–40%. The experiment was carried out until the end of the tillering phase. According to the generally accepted practice of spring wheat cultivation, treatment with Azolen^®^ and Chistalan was carried out in the tillering phase. The plants were sprayed with an aqueous emulsion (0.2 mL/plant) containing 0.5 mL/L of the herbicide, with the biopreparation (0.2 mL/plant) diluted with water to a titer of 10^8^ CFU/mL, or with solutions of the herbicide and the biopreparation mixed in equal quantities. At least 100 plants were used for each type of treatment. Fourteen days after the treatment, the fresh weight of the roots and shoots and the length of the leaves were measured. The analysis of plant tissues for hormone content and biochemical parameters was carried out on the third day after treatment of plants with Chistalan and (or) Azolen^®^.

### 4.3. Soil Climate Conditions and Experiment Design in the Field

Field experiment was carried out in 2020 and 2021 in the Baimaksky district of the Republic of Bashkortostan, Ural, Russia. The soil was Chernozem Haplic (C_org_ 3.9%, N_tot_ 0.32%, P_egner_ 143 mg/kg, K_egner_ 135 mg/kg, pH_KCl_ 6.1). The study was conducted by the method of randomized subblocks (split-plot), in three repetitions. Herbicidal treatment was not used in the first block; the second block was treated with the herbicide Chistalan. In subblocks, the biological preparation Azolen^®^ was either used or not. During the growing season in 2021–2022, the amount of precipitation was much less than the average (Table 4), so drought was a background factor during the field experiment, and there were no variants with sufficient moisture in the experimental design.

When growing plants, agricultural technology recommended for this geographical zone was used. On 6 May 2020 and 9 May 2021, 500 seeds of spring wheat of the Ekada 113 variety per square meter were sown. The predecessor was bread wheat. During the experiment, organic and mineral fertilizers were not applied. Chemical treatment of plants was carried out only with the herbicide Chistalan. In the tillering phase, the crops were sprayed once with this preparation using a manual knapsack sprayer. After the termination of tillering, the number of weeds on plots of 1 m^2^ was counted tenfold, and their species was determined. In August, all plots were harvested mechanically, with a small-plot combine harvester in the grain ripening stage. The harvest from each plot was collected separately, dried, and then weighed.

Wheat was treated with the herbicide Chistalan (0.7 L/ha), the biopreparation Azolen^®^ (2 L/ha with a titer of 10^8^ CFU/mL) at doses recommended by manufacturers, or a tank mixture of the biopreparation and herbicide. Spraying solutions were prepared by mixing preparations with water according to the manufacturer’s recommendations.

The analysis of plant tissues for relative water content, hormone content, and biochemical parameters was carried out on the fourteenth day after treatment of plants with Chistalan and (or) Azolen^®^. At the end of the experiment, the yield of grain and straw and some elements of the crop structure (dry weight of the shoot and its length, productive stems, weight of 1000 grains, and grains in the spike) were analyzed.

### 4.4. Biochemical Characteristics

All reagents used in this work (unless otherwise indicated) were manufactured by PanReac (Barcelona, Spain) and AppliChem (Darmstadt, Germany). To assess the content of the hormones indolyl-3-acetic acid (IAA), abscisic acid (ABA), and cytokinins in plant tissues, they were homogenized and extracted with 80% ethanol at +4 °C for 18 h, after which centrifugation was performed. The alcoholic extract was evaporated to an aqueous residue. To purify IAA and ABA, they were extracted with diethyl ether from an aqueous residue acidified to pH 2–3 with HCl, then transferred to a 1% NaHCO_3_ solution, and secondary re-extraction was performed with diethyl ether. The volumes of the extractants were reduced at each stage of extraction and re-extraction to free the extract from related compounds [75] and then methylated with diazomethane to stabilize the hormones in the ether fraction. After that, the ether was evaporated to dryness, and the hormones were dissolved in 0.1 mL of 80% ethanol before enzyme immunoassay. The CK contained in the aqueous residue aliquots were concentrated on a C18 column. To accomplish this, a C18 cartridge was first balanced with distilled water, and a sample that had been previously purified by centrifugation was passed through it. Then, the column was washed with 20 mL of distilled water. The cytokinin was eluted with 70% ethanol and then evaporated to dryness and dissolved in 20 mL of 80% ethanol. It was then applied to a Silica gel 60 F254 plate (Merck KGaA, Darmstadt, Germany) for thin-layer chromatography and was performed using a solvent system of butanol:ammonia:water (6:1:2) for detection of zeatin, zeatin nucleotides, and zeatin ribosides [76]. After detection, the content of each sample zone was eluted using 0.1 M of phosphate buffer (pH 7.2–7.4), and the supernatants were centrifuged at 10,000 rpm for 10 min to precipitate the silica gel.

Finally, the amount of IAA, ABA, and cytokinins was measured using solid-phase enzyme immunoassay [77]. It was performed in the wells of a polystyrene tablet. In the first stage, the hormone conjugate was sorbed on the solid phase for 1.5 h at 37 °C. After 3-fold rinsing with a saline solution containing 0.05% Tween 20, antiserum to the hormone was added to the wells, and it was diluted in a saline solution with 0.5% bovine serum albumin and 0.1% Tween 20 together with a standard hormone solution or plant extract. After an incubation period of 1 h at 30 °C, the number of specifically bound antibodies was determined using anti-rabbit IgG labeled with peroxidase (mutton antibodies). The activity of the peroxidase was assessed using a mixture containing 0.2 mg/mL of orthophenylenediamine and 0.006% hydrogen peroxide in phosphate buffer. The optical density was measured at 450 nm on a spectrophotometer (TiterTek Uniscan, Huntsville, AL, USA) after fixing the solution with sulfuric acid.

To determine the content of chlorophyll in leaves, 100 mg samples were crushed and extracted with 96% alcohol for 24 h without access to light. In the extracts, the optical density was measured at wavelengths of 665 and 649 nm. The content of chlorophylls *a* and *b* was calculated using the following formulas [78]:

C*a* = 13.7 × D_665_ − 5.76 × D_649_, C*b* = 25.8 × D_649_ − 7.6 × D_665_,

where C*a* and C*b* are the concentrations of chlorophylls *a* and *b*, and D_665_, D_649_ are the extinction values at the corresponding wavelength with subsequent conversion to the fresh weight of the sample.

Measurements were carried out in three biological and three analytical repetitions.

The content of free proline in the leaves was determined using a ninhydrin reagent, as described earlier [79], according to a calibration curve constructed with standard L-proline (Sigma-Aldrich, Burlington, MA, USA). In brief, fresh samples of the third leaves were harvested and weighted, and approximately 100 mg was used for a reaction. Then, 3% sulfosalicylic acid (5 μL/mg fresh weight) was added, and the plant material was ground using glass chips and centrifuged at 5.000× *g* rpm for 5 min (2–16 PK, Sigma, Osterode am Harz, Germany). All samples were prepared and kept on ice. The reaction mixture was prepared in a separate tube: 0.5 mL of 3% sulfosalicylic acid, 1 mL of glacial acetic acid, 1 mL of acidic ninhydrin, and 0.5 mL of the supernatant of the plant extract. The tubes were incubated at 96 °C for 60 min. The reaction was terminated on ice, and the samples were extracted with toluene. The absorbance was measured at 520 nm using toluene as reference (spectrophotometer SF-56, OKB Spectr, Saint-Petersburg, Russia)

Determination of the level of lipid peroxidation was performed using the content of malonic dialdehyde (MDA) via a method based on the formation of a colored complex between MDA and thiobarbituric acid upon heating [79]. Freshly harvested plant leaves were homogenized with distilled water, and 20% trichloroacetic acid was added to the homogenate, homogenized again, and centrifuged for 10 min at 10.000× *g* rpm. Next, equal volumes of supernatant and 0.5% thiobarbituric acid solution were added to 20% trichloroacetic acid solution, incubated at 96 °C for 30 min, and rapidly cooled in an ice bath. After centrifugation at 12.000× *g* rpm for 10 min, the optical density was determined on a spectrophotometer (SF-56, Saint-Petersburg, Russia) at wavelengths of 532 and 600 nm.

### 4.5. Characteristics of Water Relations

To determine the relative water content (RWC), the first leaf of 10 plants was taken. Selected leaves were weighed and immersed, cut in distilled water in a tightly closed vessel, and placed in the dark overnight at room temperature. A day later, the leaves were weighed again to determine the turgid weight. Then, they were dried to a constant weight, and the RWS was calculated using the following formula: relative water content = ((fresh weight − dry weight)/(turgid weight − dry weight)) × 100%.

Evapotranspiration was measured by the weight loss of pots with plants.

### 4.6. Grain Properties

The same amounts of grain from two crops were mixed to obtain averaged samples. The grain was ground in a laboratory mill with a sieve cell diameter of 0.9 mm. The grain protein content was determined by the Kjeldahl method using a nitrogen conversion factor of 5.7. To extract gluten, a dough was prepared from flour and 2% sodium chloride solution, the mass of which was 60% of the flour mass. The dough was placed in water for 40 min and then washed with running water to wash out most of the starch and obtain a clear wash water. The resulting viscoelastic mass was a wet gluten.

### 4.7. Statistical Analysis

The statistical analysis, including the Shapiro–Wilk test, was performed using the software STATISTICA version 10 (TIBCO Software Inc., Palo Alto, CA, USA). The significance of differences was assessed by ANOVA using Duncan’s test and Kruskal–Wallis test (*p* ≤ 0.05).

## 5. Conclusions

In this work, the stimulating effect of the biopreparation Azolen^®^ on wheat plants was established, which clearly manifested itself in the field experiment. Under natural drought conditions, its use helped plants overcome abiotic stress associated with a lack of moisture and herbicide spraying. This was expressed in the stabilization of water relations; a decrease in the content of MDA and ABA, as well as in the accumulation of cytokinins and auxins in the aerial parts of plants; and the increase in the activity of the photosynthetic system. All these factors together led to the increase in the growth characteristics of shoots and the improvement in the structure of the crop and grain quality. The use of the biopreparation during drought increased the grain yield by 545 kg/ha; under the same conditions, the combined use of the herbicide treatment and the biopreparation increased the yield by 1366 kg/ha. However, in order to recommend the biopreparation Azolen^®^ for inclusion in agricultural technology as an antistress agent for herbicide treatment in arid climatic zones, many more field trials on different crops and with different herbicides should be carried out.

## Figures and Tables

**Table 1:** Relative water content in wheat plants and their biochemical characteristics depending on the treatment field plots.

	Without Herbicide		Chistalan Herbicide	
	No Azolen^®^	Azolen^®^	No Azolen^®^	Azolen^®^
RWC (leaves), %	87.34 ± 1.36 ^b1^	88.65 ± 1.00 ^b^	78.50 ± 0.78 ^a^	87.91 ± 1.28 ^b^
ABA (shoots), ng/g	30.53 ^c2^	23.44 ^b^	18.08 ^a^	25.04 ^b^
IAA (shoots), ng/g	22.35 ^a2^	64.82 ^c^	81.17 ^d^	55.00 ^b^
Cytokinins (shoots), ng/g	3.43 ^a2^	6.82 ^c^	5.04 ^b^	6.54 ^c^
Chlorophyll (leaves), Mg/g	60.58 ±2.45 ^a1^	69.11 ± 3.00 ^b^	90.20 ± 5.21 ^d^	80.53 ± 3.88 ^c^
MDA, µmol/g	39.28 ± 2.42 ^b1^	26.51 ± 1.23 ^a^	40.80 ± 2.65 ^b^	25.52 ± 1.87 ^a^

^1^ Mean ± SE; different letters in each line indicate significant differences between treatments (*p* < 0.05; Duncan’s test). ^2^ Median; different letters in each line indicate significant differences between treatments (*p* < 0.05; Kruskal–Wallis test).

**Table 2:** Growth indicators, crop structure, yield, and grain quality indicators depending on the treatment field plots.

	Without Herbicide		Chistalan Herbicide	
	No Azolen^®^	Azolen^®^	No Azolen^®^	Azolen^®^
Dry weight of shoot, g	0.96 ± 0.05 ^b1^	0.95 ± 0.08 ^b^	0.71 ± 0.06 ^a^	1.24 ± 0.07 ^c^
Length of shoot, mm	265.81 ± 17.24 ^b^	299.50 ± 13.67 ^c^	230.13 ± 13.20 ^a^	315.19 ± 18.42 ^c^
Productive stems, m^−2^	389.3 ± 19.1 ^b^	521.8 ± 18.1 ^c^	357.6 ± 9.6 ^a^	604.1 ± 22.7 ^d^
Weight of 1000 grains, g	33.4 ± 1.7 ^b^	36.9 ± 1.5 ^c^	30.4 ± 1.8 ^a^	31.5 ± 1.5 ^ab^
Grains in the spike	16.9 ± 0.8 ^a^	18.9 ± 0.3 ^b^	19.3 ± 0.7 ^b^	20.8 ± 0.4 ^c^
Yield, kg/ha	2180 ± 143 ^a^	2725 ± 132 ^b^	2090 ± 124 ^a^	3456 ± 128 ^c^
Straw, kg/ha	2200 ± 38 ^a^	3762 ± 88 ^c^	2420 ± 40 ^b^	4421 ± 29 ^d^
Gluten, %	39.32 ± 1.00 ^a^	42.98 ± 1.11 ^b^	39.72 ± 0.89 ^a^	42.74 ± 1.13 ^b^
Protein, %	13.94 ± 0.74 ^b^	14.12 ± 0.80 ^b^	11.39 ± 0.71 ^a^	14.00 ± 0.82 ^b^

^1^ Mean ± SE; different letters in each line indicate significant differences between treatments (*p* < 0.05; Duncan’s test).

**Table 3:** Variants of treatment in a laboratory experiment.

Variable Parameters	Variant							
	Control	Ch	A	Ch + A	Control Drought	Ch Drought	A Drought	Ch + A Drought
Normal hydration	+	+	+	+	−	−	−	−
Moisture deficit	−	−	−	−	+	+	+	+
Chistalan (Ch)	−	+	−	+	−	+	−	+
Azolen^®^ (A)	−	−	+	+	−	−	+	+

+ indicates the presence of the parameter, − indicates the absence of the parameter.

**Table 4:** Average climatic indicators of the cropping seasons of 2020 and 2021.

	May	June	July	August
	Rainfall, mm			
2020	24.0 ± 1.2	0	32.7 ± 1.9	15.3 ± 0.9
2021	0.4 ± 0.01	26.2 ± 1.1	0.5 ± 0.01	4.4 ± 0.3
Average long-term	29.1 ± 1.4	43.9 ± 2.3	49.4 ± 3.0	44.1 ± 2.5
	**Average temperature, °C**			
2020	+16.0 ± 1.1	+18.0 ± 1.1	+23.1 ± 1.7	+20.1 ± 1.5
2021	+17.2 ± 1.0	+19.6 ± 1.3	+19.5 ± 0.8	+22.2 ± 1.4
Average long-term	+12.5 ± 0.8	+16.8 ± 0.9	+18.5 ± 1.0	+16.4 ± 0.7
**Average humidity** **, %**				
2020	52	46	53	40
2021	41	44	47	38

## Data Availability

The data presented in this study are available in the graphs and tables provided in the manuscript.
